# Association between the survey-based women’s empowerment index (SWPER) and intimate partner violence in sub-Saharan Africa

**DOI:** 10.1186/s12978-024-01755-8

**Published:** 2024-05-10

**Authors:** Irene Esi Donkoh, Richard Gyan Aboagye, Joshua Okyere, Abdul-Aziz Seidu, Bright Opoku Ahinkorah, Sanni Yaya

**Affiliations:** 1 Department of Medical Laboratory Science, University of Cape Coast, Cape Coast, Ghana; 2 Department of Family and Community Health, Fred N. Binka School of Public Health, University of Health and Allied Sciences, Hohoe, Ghana; 3 Department of Population and Health, University of Cape Coast, Cape Coast, Ghana; 4 School of Nursing and Midwifery, College of Health Sciences, Kwame Nkrumah University of Science and Technology, Kumasi, Ghana; 5 Centre for Gender and Advocacy, Takoradi Technical University, Takoradi, Ghana; 6 College of Public Health, Medical and Veterinary Sciences, James Cook University, Townsville, Australia; 7 REMS Consultancy Services Limited, Sekondi-Takoradi, Western region Ghana; 8 School of Clinical Medicine, University of New South Wales, Sydney, Australia; 9 School of International Development and Global Studies, University of Ottawa, Ottawa, Canada; 10 The George Institute for Global Health, Imperial College London, London, UK

**Keywords:** Emotional violence, Intimate partner violence, Physical violence, Sexual violence, SWPER

## Abstract

**Background:**

Intimate partner violence (IPV) is high among women of reproductive age in sub-Saharan Africa (SSA). However, empowering women enables them to confront and mitigate IPV. In this study, we examined the association between the survey-based women's empowerment index (SWPER) and IPV in SSA.

**Methods:**

We used data from the Demographic and Health Surveys of 19 countries conducted from 2015 to 2021. Our study was restricted to a weighted sample of 82,203 women of reproductive age who were married or cohabiting. We used spatial maps to show the proportions of women who experienced past-year IPV. A five-modelled multilevel binary logistic regression analysis was adopted to examine the association between SWPER and IPV. The results were presented using the adjusted odds ratio (AOR) with their respective 95% confidence interval (CI). Statistical significance was set at p < 0.05.

**Results:**

With physical and emotional violence, the country with the highest prevalence was Sierra Leone, with a prevalence of 39.00% and 38.97% respectively. Rwanda (10.34%), Zambia (11.09%), Malawi (15.00%), Uganda (16.88%), and Burundi (20.32%) were the hotspot countries for sexual violence. Angola (34.54%), Uganda (41.55%), Liberia (47.94%), and Sierra Leone (59.98%) were the hotspot countries for IPV. A high SWPER score in attitudes to violence significantly decreased the odds of IPV [AOR = 0.70; 95% CI = 0.66, 0.75]. Also, women with medium score in decision-making were less likely to experience IPV compared to those with lower scores [AOR = 0.89; 95% CI = 0.83, 0.95]. However, higher odds of experiencing IPV was found among women with medium score in autonomy compared to those with low scores [AOR = 1.07; 95% CI = 1.01, 1.14].

**Conclusions:**

Our study has shown that the three dimensions of SWPER significantly predict IPV among women. Consequently, it is crucial that sub-Saharan African countries implement various initiatives, such as IPV advocacy programs and economic livelihood empowerment initiatives. These initiatives should not only aim to improve women's attitudes to domestic violence but also to enhance their social independence, autonomy, and decision-making capacity.

**Supplementary Information:**

The online version contains supplementary material available at 10.1186/s12978-024-01755-8.

## Background

Achieving gender equality and empowering all women and girls is the aim of the United Nations Sustainable Development Goal (SDG) 5. In accordance with this, SDG 5.2 advocates for the abolition of all forms of violence against women and girls in both public and private domains, including trafficking, sexual and other forms of exploitation [1]. However, gender inequality persists, causing over 8 million disability-adjusted life years (DALYs), more than 4 million years lost due to disability (YLD), over 80 thousand fatalities, and sexually transmitted infections due to sexual exploitation [2, 3]. This barrier of gender inequality is the fundamental cause of violence against women, which poses a risk to public health, violating human rights, and impeding national progress. This violence manifests in diverse forms with one in three of all women worldwide having experienced physical, emotional, or sexual abuse at the hands of an intimate partner [4]. The most common type of violence is intimate partner violence (IPV), which is reported by 30% of women who have been in a relationship [4]. The mental and physical health implications and human rights abuse on IPV survivors is alarming [1, 5].

IPV is prevalent in most countries worldwide, with variations between countries [6, 7]. Available evidence indicates that the burden of IPV ranges from 37% in least developed countries to 16–23% and 18–21% in Europe and Central, Eastern and South-Eastern Asia respectively [4]. In the context of sub-Saharan Africa (SSA), the experience of IPV is higher among women of reproductive age [8, 9] with a prevalence of 33% which is generally higher than the global average [4].

Studies argue that women's empowerment can provide women with the autonomy and power to mitigate IPV through education, enabling them to know their place in society. However, this assertion seems futile [10, 11], as some empowered women still experience IPV. To measure this empowerment in terms of socioeconomic, health disparity and gender safety, particularly for marginalized gender groups, several indices such as the Gender Development Index, Global Gender Gap Index, Social Institutions and Gender Index, the Gender Inequality Index, Peace and Security Index developed in recent years [12–15]. A survey-based women's empowerment (SWPER) was developed and validated using Demographic and Health Surveys (DHS) data from 34 African countries [16]. Recognising the importance of these sub countries, SWPER was developed to curb this challenge [17, 18]. Among partnered (married or in a union) women, SWPER measures three empowerment domains (social independence, decision-making, and attitude to violence) that are indicative of assets and agency [16–18]. SWPER was developed using a conceptual framework that is comparable to a recently proposed one that identifies three types of empowerment: intrinsic, instrumental, and enabling factors [18, 19].

SWPER employs individual-level data to enable the assessment of relationships between empowerment, various health interventions, and outcomes [16–18]. SWPER also enables periodic analysis of within-country and between-country comparisons. Over 60 nations with DHS have access to the data needed to calculate the SWPER [16, 17]. While the meaning of women's empowerment and autonomy may be of different views to many, it however does not relegate the fact that IPV must be curbed. It has therefore become more necessary to use SWPER to evaluate this and put an end to this contradiction. Such that, crucial and rapt interventions to improve, promote and maintain the health of women and give them their rightful place in society. As such, this paper seeks to address the existing conflicting ideologies and to empower women in this heightened era of gender equality and changes in gender roles. We, therefore, examined the association between the dimensions of SWPER and experience of IPV in SSA.

## Methods

### Data source and study design

We sourced data from the DHS of nineteen countries in SSA, spanning from 2015 to 2021. The data used were extracted from the DHS Program, which is available upon request [20]. We have provided the list of the countries and their survey years in Table 1. Since the inception of DHS, there have been more than 400 surveys conducted in over 90 low-and middle-income countries [21]. A cross-sectional design was used for the DHS. The respondents were sampled using a multistage sampling technique with the detailed sampling methodology highlighted in the literature [21, 22]. Our study was restricted to a weighted sample of 82,203 women in their reproductive age who were married or cohabiting. We followed the Strengthening the Reporting of Observational Studies in Epidemiology (STROBE) guidelines in writing this paper [23].

**Table 1:** Description of study sample per country

S1Country	Year of survey	Weighted sample	Weighted percentage
1. Angola	2015–16	4859	5.91
2. Benin	2017–18	4291	5.22
3. Burundi	2016–17	4400	5.35
4. Cameroon	2018	3921	4.77
5. Ethiopia	2016	4370	5.32
6. Gambia	2019–20	3038	3.70
7. Liberia	2019–20	1939	2.36
8. Madagascar	2021	5054	6.15
9. Mali	2018	2984	3.63
10. Malawi	2015–16	6518	7.93
11. Nigeria	2018	11,515	14.01
12. Rwanda	2019–20	3696	4.50
13. Sierra Leone	2019	4023	4.89
14. Chad	2014–15	4749	5.78
15. Tanzania	2015–16	3542	4.31
16. Uganda	2016	4784	5.82
17. South Africa	2016	2264	2.75
18. Zambia	2018	3531	4.30
19. Zimbabwe	2015	2725	3.31
All countries	2015–2021	82,203	100.00

### Variables

There were four outcome variables in this study. The first three were past-year experiences of physical, emotional, and sexual violence from a partner or husband. Physical, emotional, and sexual violence were derived from the modified Conflict Tactics Scale [24, 25], a list of questions used to measure the extent to which individuals in sexual relationship experience physical, emotional, and sexual violence. In the DHS, women in sexual unions: married or cohabiting were asked to indicate whether they have experienced any physical, emotional, and sexual violence in the last 12 months preceding the survey. The fourth outcome variable was created from a composite of physical, emotional, and sexual violence. This was referred to as IPV. Specific questions used to measure physical, emotional, and sexual violence are available in the literature that used the DHS dataset [26–29]. We also used the existing coding of physical, emotional, and sexual violence guided by previous studies [26–29].

We used the newly developed and validated SWPER as the key explanatory variable. It was statistically created for use in low-and middle-income countries [16]. Since its emergence, SWPER has been used to address several health and social issues, including reproductive health, maternal and child health, and other related topics [16–18]. SWPER was developed using fourteen variables. The variables consisted of (i) beating not justified if wife goes out without telling husband, (ii) beating not justified if wife neglects the children, (iii) beating not justified if wife argues with husband, (iv) beating not justified if wife refuses to have sex with husband, (v) beating not justified if wife burns the food, (vi) frequency of reading newspaper or magazine, (vii) woman education, (viii) age of respondent at cohabitation, (ix) age of respondent at first birth, (x) age difference: woman’s age minus husband’s age, (xi) education difference: woman’s minus husband’s years of schooling, (xii) who usually decides on respondent's health care, (xiii) who usually decides on large household purchases, and (xiv) who usually decides on visits to family or relatives [16]. These fourteen variables were used to create the dimensions of SWPER [16]. The three dimensions are attitude to violence, social autonomy, and decision-making. Social independence or autonomy denotes the preconditions such as the schooling attainment, information access, age at crucial life events, and spousal asset differentials that allow women to realize their goals. Decision-making on the other hand refers to the degree of the woman's involvement in household decisions, which can also be viewed as a gauge of instrumental agency. Finally, attitude to violence closely related to the concept of intrinsic agency and it is a proxy for the woman’s incorporation of gender norms-related to the acceptability of IPV [16]. We used the same coding methodology as used in the previous study conducted by Ewerling et al. [16]. For attitude to violence, the coding for each category consisted of low (score ≤ − 0.700), medium (score > − 0.700 ≤ 0.400), and high (score > 0.400). The high category denotes strong disagreement or rejection of attitude to violence (positive), whereas the low group emphasizes strong acceptance of violence (negative). Low (score ≤ − 0.559), medium (score > − 0.559 ≤ 0.293), and high (score > 0.293) were the coding and classification of the social independence dimension. Whereas, those of decision-making were low (score ≤ − 1.000), medium (score > − 1.000 ≤ 0.600), and high (score > 0.600) [16].

We included six covariates in our study. These covariates either increase or decrease women’s likelihood of experiencing IPV based on literature [26–29]. Also, the covariates were present in the DHS dataset across all the countries included in the study. The covariates were grouped into individual and contextual level variables. The individual level variables consisted of partner alcohol consumption, exposure to interparental violence, and exposure to partner controlling behavior. Likewise, household wealth index, place of residence, and geographical sub-regions were the contextual level variables.

### Statistical analyses

We used Stata version 17.0 (Stata Corporation, College Station, TX, USA) to perform all the analyses. We carried out data cleaning and weighting at the country level per the DHS guidelines before appending the dataset for final analysis. To do this, the weighting variable for domestic violence module (d005) was divided by 1,000,000 to generate a new variable called “= d005_pw”. Next, we de-normalized the country level weights using the command: gen d005_pwpool = d005_pw*(total population of women; age 15–49 at the time of the survey/number of women in the resulting domestic violence subsample. Later, we appended the weighted country-level dataset for the 19 countries and used for the final analysis. We used ‘spmap’ in Stata to generate the proportion of women who experienced physical, emotional, sexual violence, and IPV in the past prior to the survey. We examined the distribution of the outcome variables across the dimensions of SWPER, and the covariates, as well as their associations using Pearson's Chi-square test. This was followed by a five-modelled multilevel binary logistic regression modelling. Prior to the regression analysis, we checked for evidence of multicollinearity among the variables using the variance inflation factor (VIF). The results showed that the minimum, maximum, and mean VIFs were 1.04, 3.48, and 2.00, respectively. Hence, there was no evidence of high collinearity among the variables. The first model had no explanatory variables or covariates, showing the variance in the outcome variables attributed to the primary sampling units (PSU). Model I was fitted to contain the three dimensions of SWPER. Model II contained the variables in Model I and the individual-level covariates. The variables in Model II and the contextual level covariates were placed in Model III. The final model (Model IV) contained the dimensions of SWPER and all the covariates. The results were presented using adjusted odds ratio (aOR) with their respective 95% confidence interval (CI). Statistical significance was set at p < 0.05.

### Ethical consideration

Ethical clearance was not sought for this study since we analyzed a secondary dataset, which is already available freely to use. We obtained permission to use the dataset from the DHS program data repository before using the dataset for publication.

## Results

### Prevalence of intimate partner violence among women in sub-Saharan Africa

**Fig. 1:**
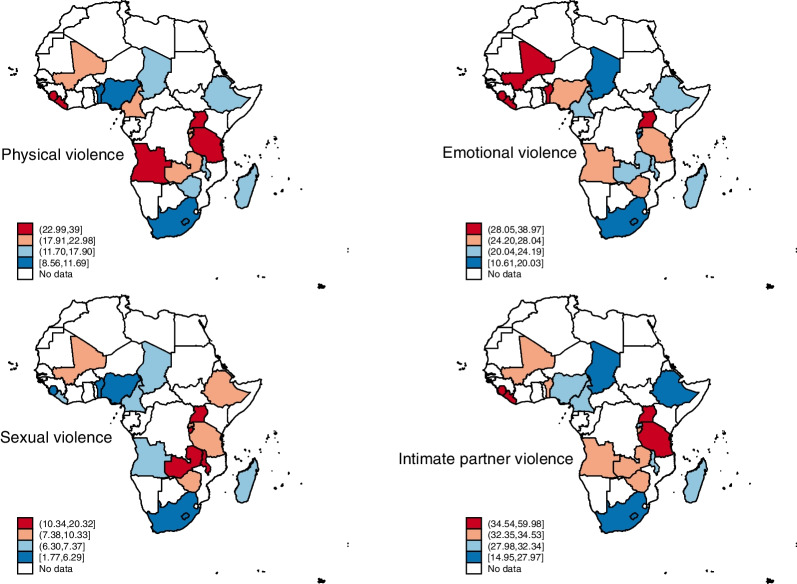
Proportion of physical violence, emotional violence, sexual violence, and IPV across the 19 countries in SSA

The proportion of physical violence, emotional violence, sexual violence, and IPV across the 19 countries in SSA have been presented in Fig. 1. With physical violence, the countries with the highest proportions of IPV were Uganda (22.99%), Angola (24.14%), Tanzania (26.57%), Liberia (35.81%), and Sierra Leone (39%). For emotional violence, Mali (28.05%), Benin (29.23%), Uganda (30.81%), Liberia (36.44%), and Sierra Leone (38.97%) had the highest proportions of IPV. Rwanda (10.34%), Zambia (11.09%), Malawi (15.00%), Uganda (16.88%), and Burundi (20.32%) were the countries with the highest proportions of sexual violence. Angola (34.54%), Uganda (41.55%), Liberia (47.94%), and Sierra Leone (59.98%) were the countries with the highest proportions of IPV (see Additional file 1: Table S1 for full prevalence).

### Distribution of intimate partner violence across the explanatory variables

Table 2 shows the distribution of IPV across the dimensions of SWPER and the covariates. There were observable significant differences in physical violence across the SWPER dimensions with the highest proportions being observed among women with low scores on the attitude to violence scale (23.8%), those who had medium scores on the social independence (autonomy) (19.8%), and those who scored high on the decision-making scale (19.0%). For emotional violence, the highest proportions were recorded among women with low attitude towards violence (28.7%), those with moderate social independence (25.6%), whilst those in the low and high categories of decision-making reporting the same proportion (25.8%). Also, the highest prevalence of sexual violence was found among women who had low attitude towards violence (11.3%), those with moderate social independence (10.5%), and those with high decision-making (9.4%). For IPV (experiencing at least one of physical, emotional, and sexual violence), the highest proportions were reported among women with low attitude towards violence (38.7%), women with medium social independence (34.6%), and women with low decision-making (33.4%). All the variables showed statistically significant associations with physical violence, emotional violence, sexual violence, and IPV at p < 0.05.

**Table 2:** Distribution of intimate partner violence across the explanatory variables

Variable	Weighted n (%)	Physical violence	p-value	Emotional violence	p-value	Sexual violence	p-value	IPV	p-value
Attitude to violence			< 0.001		< 0.001		< 0.001		< 0.001
Low	21,316 (25.9)	23.8		28.7		11.7		38.7	
Medium	14,462 (17.6)	21.5		27.5		11.1		37.5	
High	46,425 (56.5)	14.1		21.3		6.8		27.3	
Social independence (autonomy)			< 0.001		< 0.001		< 0.001		< 0.001
Low	25,050 (30.5)	18.0		24.7		8.3		32.2	
Medium	28,588 (34.8)	19.8		25.6		10.5		34.6	
High	28,565 (34.7)	16.1		22.7		7.7		29.4	
Decision-making			< 0.001		< 0.001		0.029		< 0.001
Low	16,199 (19.7)	18.8		25.8		8.8		33.4	
Medium	38,678 (47.1)	16.9		22.6		8.5		30.6	
High	27,326 (33.2)	19.0		25.8		9.4		33.3	
Partner alcohol consumption			< 0.001		< 0.001		< 0.001		< 0.001
No	54,485 (66.3)	12.8		19.1		6.2		25.4	
Yes	27,718 (33.7)	28.2		34.5		14.0		45.1	
Exposed to interparental violence			< 0.001		< 0.001		< 0.001		< 0.001
No	63,465 (77.2)	14.7		21.2		7.2		27.7	
Yes	8738 (22.8)	29.2		34.7		14.5		46.6	
Experienced partner controlling behavior			< 0.001		< 0.001		< 0.001		< 0.001
No	31,154 (37.9)	6.6		8.8		3.4		14.0	
Yes	51,049 (62.1)	24.9		33.8		12.2		43.1	
Wealth index			< 0.001		< 0.001		< 0.001		< 0.001
Poorest	15,843 (19.3)	19.9		25.5		9.5		34.1	
Poorer	16,653 (20.2)	20.0		25.5		10.1		34.2	
Middle	16,749 (20.4)	19.1		25.1		9.6		33.6	
Richer	16,493 (20.1)	17.6		24.8		9.0		32.4	
Richest	16,465 (20.0)	13.2		20.6		5.9		25.9	
Place of residence			0.003		0.010		< 0.001		< 0.001
Urban	27,534 (33.5)	16.9		23.3		6.5		29.5	
Rural	54,669 (66.5)	18.5		24.8		10.0		33.3	
Geographical subregions			0.001		< 0.001		< 0.001		< 0.001
Central Africa	13,529 (16.4)	19.4		20.5		6.7		29.3	
Southern Africa	8520 (10.4)	15.9		20.1		8.5		27.8	
Eastern Africa	32,363 (39.4)	18.3		23.5		12.9		33.0	
Western Africa	27,790 (33.8)	17.5		28.4		5.3		33.5	

P-values were generated from the Pearson chi-square test

### Association between the dimensions of SWPER index and physical violence

Table 3 shows the results of the association between the dimensions of SWPER and physical violence, controlling for the covariates. The results showed that the odds of experiencing physical violence decreases with decreasing attitude towards violence (rejecting violence towards women) with the lowest odds among those with high attitude towards violence [AOR = 0.62; 95% CI = 0.57, 0.66]. Women who had medium autonomy were more likely to experience physical violence [AOR = 1.08; 95% CI = 1.01, 1.15] compared to those who had low autonomy.  Compared to women who scored low in decision-making, those who had medium scores [AOR = 0.88; 95% CI = 0.81, 0.96] were less likely to experience physical violence.

**Table 3:** Association between dimensions of SWPER and physical violence

Variable	Model O	Model I AOR [95% CI]	Model II AOR [95% CI]	Model III AOR [95% CI]	Model IV AOR [95% CI]
*Fixed effect model*					
Attitude to violence					
Low		1.00	1.00	1.00	1.00
Medium		0.87^***^ [0.81, 0.94]	0.88^**^ [0.81, 0.96]	0.88^***^ [0.82, 0.95]	0.90^**^ [0.83, 0.98]
High		0.53^***^ [0.50, 0.57]	0.61^***^ [0.56, 0.65]	0.54^***^ [0.50, 0.58]	0.62^***^ [0.57, 0.66]
Social independence (autonomy)					
Low		1.00	1.00	1.00	1.00
Medium		1.12^***^ [1.05, 1.19]	1.03 [0.97, 1.10]	1.14^***^ [1.07, 1.22]	1.08* [1.01, 1.15]
High		0.95 [0.88, 1.02]	0.86^***^ [0.80, 0.93]	1.03 [0.96, 1.12]	0.95 [0.88, 1.03]
Decision-making					
Low		1.00	1.00	1.00	1.00
Medium		0.93 [0.86, 1.01]	0.82^***^ [0.75, 0.89]	0.94 [0.87, 1.02]	0.88^*^ [0.81, 0.96]
High		1.14^**^ [1.04, 1.25]	0.87^**^ [0.80, 0.96]	1.19^***^ [1.08, 1.30]	0.98 [0.0.89, 1.08]
Random effect results					
PSU variance (95% CI)	0.896 [0.741, 1.083]	0.813 [0.670, 0.99]	0.688 [0.564, 0.839]	0.792 [0.653, 0.962]	0.699 [0.573, 0.854]
ICC	0.214	0.198	0.173	0.194	0.175
Wald chi-square	Reference	542.36 (< 0.001)	3351.75 (< 0.001)	646.26 (< 0.001)	3577.14 (< 0.001)
Model fitness					
Log-likelihood	− 127,996.57	− 126,181.51	− 112,963.54	− 125,657.73	− 112,280.87
AIC	255,997.1	252,379	225,949.1	251,347.5	224,599.7
N	82,203	82,203	82,203	82,203	82,203
Number of clusters	1395	1395	1395	1395	1395

aOR: adjusted odds ratios; CI: confidence interval; ^*^p < 0.05, ^**^p < 0.01, ^***^p < 0.001; 1.00 = Reference category; PSU: primary sampling unit; ICC: intra-class correlation coefficient; AIC: Akaike’s information criterion

Model I = Included only the dimensions of SWPER

Model II = Included variables in Model I and partner alcohol use, exposure to interparental violence, and experience of partner controlling behaviour

Model III = Included variables in Model I and wealth index, place of residence, and sub-region

Model IV = Included variables in Model II and wealth index, place of residence, and sub-region

### Association between the dimensions of SWPER and emotional violence

Two out of the three dimensions of SWPER were significantly associated with emotional violence. Specifically, lower risk of emotional violence was observed among women who scored high in attitude to violence [AOR = 0.79; 95% CI = 0.74, 0.85] compared to those who had low scores. Also, the odds of experiencing emotional violence was lower among women who had medium decision-making score compared to those with low scores [AOR = 0.90; 95% CI = 0.84, 0.97] (Table 4).

**Table 4:** Association between the dimensions of SWPER and emotional violence

Variable	Model O	Model I AOR [95% CI]	Model II AOR [95% CI]	Model III AOR [95% CI]	Model IV AOR [95% CI]
*Fixed effect model*					
Attitude to violence					
Low		1.00	1.00	1.00	1.00
Medium		0.94 [0.88, 1.01]	0.96 [0.89, 1.04]	0.96 [0.89, 1.03]	0.99 [0.92, 1.07]
High		0.66^***^ [0.62, 0.71]	0.78^***^ [0.73, 0.83]	0.68^***^ [0.64, 0.72]	0.79^***^ [0.74, 0.85]
Social independence (autonomy)					
Low		1.00	1.00	1.00	1.00
Medium		1.05 [0.99, 1.11]	0.98 [0.92, 1.04]	1.09^**^ [1.03, 1.15]	1.02 [0.96, 1.09]
High		0.93^*^ [0.87, 0.99]	0.86^***^ [0.80, 0.92]	1.01 [0.95, 1.08]	0.95 [0.88, 1.02]
Decision-making					
Low		1.00	1.00	1.00	1.00
Medium		0.86^***^ [0.80, 0.92]	0.79^***^ [0.73, 0.85]	0.94 [0.88, 1.01]	0.90^**^ [0.84, 0.97]
High		1.07 [0.99, 1.16]	0.87^***^ [0.51, 0.94]	1.26^***^ [1.16, 1.36]	1.08 [0.99, 1.17]
Random effect results					
PSU variance (95% CI)	0.622 [0.505, 0.767]	0.609 [0.496, 0.749]	0.536 [0.436, 0.658]	0.636 [0.516, 0.784]	0.532 [0.429, 0.658]
ICC	0.159	0.156	0.140	0.162	0.139
Wald chi-square	Reference	302.49 (< 0.001)	3478.20 (< 0.001)	482.57 (< 0.001)	4051.33 (< 0.001)
Model fitness					
Log-likelihood	− 151,687.61	− 150,540.06	− 134,641.02	− 149,434.06	− 132722.46
AIC	303,379.2	301,096.1	269,304.0	298,900.1	265,482.9
N	82,203	82,203	82,203	82,203	82,203
Number of clusters	1395	1395	1395	1395	1395

aOR: adjusted odds ratios; CI: confidence interval; ^*^p < 0.05, ^**^p < 0.01, ^***^p < 0.001; 1.00 = Reference category; PSU: primary sampling unit; ICC: intra-class correlation coefficient; AIC: Akaike’s information criterion

Model I = Included only the dimensions of SWPER

Model II = Included variables in Model I and partner alcohol use, exposure to interparental violence, and experience of partner controlling behaviour

Model III = Included variables in Model I and wealth index, place of residence, and sub-region

Model IV = Included variables in Model II and wealth index, place of residence, and sub-region

### Association between the dimensions of SWPER and sexual violence

All three dimensions of SWPER were significantly associated with sexual violence. Compared to those who scored low on the attitude to violence scale, there was a significantly lower risk of sexual violence among women with medium [AOR = 0.90; 95% CI = 0.82, 0.99] and higher scores [AOR = 0.66; 95% CI = 0.60, 0.72]. A higher likelihood of sexual violence was observed among women who had medium scores in social independence [AOR = 1.13; 95% CI = 1.03, 1.24] compared to those with low scores. Having medium [AOR = 0.74; 95% CI = 0.66, 0.82] and higher scores [AOR = 0.78; 95% CI = 0.71, 0.90] in decision-making were less likely to experience sexual violence compared to those with low scores in decision-making (see Table 5).

**Table 5:** Association between the dimensions of SWPER and sexual violence

Variable	Model O	Model I AOR [95% CI]	Model II AOR [95% CI]	Model III AOR [95% CI]	Model IV AOR [95% CI]
*Fixed effect model*					
Attitude to violence					
Low		1.00	1.00	1.00	1.00
Medium		0.91 [0.83, 1.00]	0.93 [0.84, 1.02]	0.88^**^ [0.80, 0.97]	0.90^*^ [0.82, 0.99]
High		0.54^***^ [0.50, 0.59]	0.63^***^ [0.58, 0.69]	0.57^***^ [0.52, 0.62]	0.66^***^ [0.60, 0.72]
Social independence (autonomy)					
Low		1.00	1.00	1.00	1.00
Medium		1.31^***^ [1.21, 1.43]	1.23^***^ [1.12, 1.34]	1.16^***^ [1.07, 1.27]	1.13^*^ [1.03, 1.24]
High		1.02 [0.93, 1.11]	0.93 [0.85, 1.02]	0.96 [0.88, 1.06]	0.93 [0.84, 1.02]
Decision-making					
Low		1.00	1.00	1.00	1.00
Medium		1.02 [0.92, 1.13]	0.91 [0.82, 1.01]	0.76^***^ [0.69, 0.85]	0.74^***^ [0.66, 0.82]
High		1.20^**^ [1.07, 1.35]	0.94 [0.84, 1.06]	0.91 [0.81, 1.02]	0.78^***^ [0.69, 0.88]
Random effect model					
PSU variance (95% CI)	0.773 [0.642, 0.931]	0.684 [0.568, 0.823]	0.603 [0.496, 0.733]	0.603 [0.493, 0.736]	0.565 [0.458, 0.698]
ICC	0.190	0.172	0.155	0.155	0.147
Wald chi-square	Reference	336.09 (< 0.001)	2045.68 (< 0.001)	805.94 (< 0.001)	2347.89 (< 0.001)
Model fitness					
Log-likelihood	− 81,281.036	− 80,219.986	− 74,564.76	− 78,185.911	− 72,985.632
AIC	162,566.1	160,456	149,151.5	156,403.8	146,009.3
N	82,203	82,203	82,203	82,203	82,203
Number of clusters	1395	1395	1395	1395	1395

aOR: adjusted odds ratios; CI: confidence interval; ^*^p < 0.05, ^**^p < 0.01, ^***^p < 0.001; 1.00 = Reference category; PSU: primary sampling unit; ICC: intra-class correlation coefficient; AIC: Akaike’s information criterion

Model I = Included only the dimensions of SWPER

Model II = Included variables in Model I and partner alcohol use, exposure to interparental violence, and experience of partner controlling behaviour

Model III = Included variables in Model I and wealth index, place of residence, and sub-region

Model IV = Included variables in Model II and wealth index, place of residence, and sub-region

### Association between the dimensions of SWPER and intimate partner violence

Table 6 presents the results of the association between the dimensions of SWPER and IPV. High SWPER score in attitudes towards violence significantly decreases the odds of IPV [AOR = 0.70, 95% CI = 0.66, 0.75]. Women with medium score in decision-making [AOR = 0.89; 95% CI = 0.83, 0.96] were less likely to experience IPV relative to those with low scores. However, higher odds of experiencing IPV was found among women with medium score in autonomy compared to those with low scores [AOR = 1.07; 95% CI = 1.01, 1.14].

**Table 6:** Association between the dimensions of SWPER and intimate partner violence

Variable	Model O	Model I AOR [95% CI]	Model II AOR [95% CI]	Model III AOR [95% CI]	Model IV AOR [95% CI]
*Fixed effect model*					
Attitude to violence					
Low		1.00	1.00	1.00	1.00
Medium		0.95 [0.89, 1.01]	0.97 [0.90, 1.04]	0.96 [0.90, 1.03]	0.99 [0.93, 1.07]
High		0.59^***^ [0.56, 0.63]	0.68^***^ [0.64, 0.73]	0.61^***^ [0.58, 0.65]	0.70^***^ [0.66, 0.75]
Social independence (autonomy)					
Low		1.00	1.00	1.00	1.00
Medium		1.11^***^ [1.05, 1.18]	1.03 [0.97, 1.09]	1.13^***^ [1.07, 1.20]	1.07* [1.01, 1.14]
High		0.94 [0.88, 1.00]	0.86^***^ [0.80, 0.92]	1.02 [0.96, 1.08]	0.95 [0.88, 1.01]
Decision-making					
Low		1.00	1.00	1.00	1.00
Medium		0.91^**^ [0.85, 0.97]	0.82^***^ [0.76, 0.88]	0.94 [0.88, 1.01]	0.89^**^ [0.83, 0.96]
High		1.09^*^ [1.01, 1.17]	0.86^***^ [0.79, 0.93]	1.19^***^ [1.10, 1.29]	1.00 [0.92, 1.09]
Random effect model					
PSU variance (95% CI)	0.674 [0.549, 0.827]	0.626 [0.510, 0.768]	0.499 [0.406, 0.614]	0.639 [0.520, 0.785]	0.519 [0.421, 0.640]
ICC	0.170	0.160	0.132	0.163	0.136
Wald chi-square	Reference	546.29 (< 0.001)	5086.18 (< 0.001)	648.16 (< 0.001)	5581.17 (< 0.001)
Model fitness					
Log-likelihood	− 171,225.41	− 169,219.6	− 149,889.08	− 168,502.41	− 148,633.6
AIC	342,454.8	338,455.2	299,800.2	337,036.8	297305.2
N	82,203	82,203	82,203	82,203	82,203
Number of clusters	1395	1395	1395	1395	1395

aOR: adjusted odds ratios; CI: confidence interval; ^*^p < 0.05, ^**^p < 0.01, ^***^p < 0.001; 1.00 = Reference category; PSU: primary sampling unit; ICC: intra-class correlation coefficient; AIC: Akaike’s information criterion; Model I=Included only the dimensions of SWPER; Model II= Included variables in Model I and partner alcohol use, exposure to interparental violence, and experience of partner controlling behaviour; Model III = Included variables in Model I and wealth index, place of residence, and sub-region; and Model IV = Included variables in Model II and wealth index, place of residence, and sub-region

## Discussion

The SDG 5 emphasizes the importance of achieving gender equality and empowerment of women and girls. To achieve this, there is a need for evidence-based research. Therefore, we examined the association between the dimension of SWPER and IPV among women in SSA.

Our study demonstrates that the distribution of IPV differs between the various countries included in the study. Notably, Sierra Leone consistently emerged as a hotspot with the highest proportion of IPV while South Africa had the least proportion. The observed prevalence of IPV in South Africa is consistent with a previous study that found similar findings [26]. However, a study by Horn et al. [27] suggest that the existence of “a poorly functioning criminal justice system and a social system in which children often stay with fathers following separation or divorce” may explain the high prevalence of IPV in Sierra Leone. A qualitative study [28] also opines that conflicts in Sierra Leone may have facilitated men’s normalization of resorting to violence in resolving frustration in intimate relationships. Hence, the high prevalence of physical and emotional violence in Sierra Leone. Other countries that emerged as hotspots (i.e., areas with high prevalence of IPV) for IPV were Uganda, Liberia, and Angola. All of these countries have been victims of civil wars and conflicts in the past. In the case of Angola, it was not until 2011 that the country categorized IPV as a crime [29].

All three dimensions of SWPER were significantly associated with IPV. A higher SWPER score in the domain of decision-making was associated with lower odds of IPV. At the individual typologies of IPV, this pattern of association was true for the risk of experiencing physical, sexual and emotional violence. The result corroborates studies conducted in Ethiopia [30] and Ghana [31] that have shown that the risk of IPV is significantly reduced when women have a higher decision-making capacity. This association could be that higher decision-making tends to promote open communication, compromise, and problem-solving which reduces the likelihood of conflicts escalating into violence within an intimate partner relationship. It is also possible that women who actively participate in decision-making challenge traditional gender norms and stereotypes that perpetuate unequal power dynamics [32]. This challenges the underlying attitudes that can contribute to abusive behavior.

Although not statistically significant, we found that women who scored high on autonomy were less likely to experience IPV compared to those who had low scores. However, women with higher scores in autonomy were less likely to experience IPV, though not significant. Nonetheless, statistically significant associations have been reported in previous studies [33–35]. This is consistent with Bengesai and Khan who [33] found that low levels of autonomy increased the risk of IPV by 1.5 folds. Kebede et al. [35] also revealed that the risk of IPV was 82% lower among women with high autonomy. This implies that advancing women’s autonomy could be critical to alleviating the incidence of IPV in SSA. As indicated by Tenkorang [31], autonomous women tend to be more educated. The implication is that such women are more likely to seek out information and education on topics like healthy relationships, gender dynamics, and violence prevention. This knowledge can lead to a better understanding of warning signs and potential risks associated with abusive relationships. As a result, autonomous women may be more likely to recognize signs of IPV and take proactive steps to address or avoid such situations. The results, however, showed that moderate scores in the autonomy scale was associated with higher likelihood of experiencing IPV compared to those with low scores.

Having higher scores in the SWPER domain of attitudes to violence was associated with the risk of IPV among women. Thus, women who were highly intolerant of violence were less likely to experience IPV. Similar patterns of association were observed across physical, emotional, and sexual violence. The result is inconsistent with Copp et al.’s [36] study that found the likelihood of IPV to be high among those who had favorable attitudes towards domestic violence, which could partly be due to the differences in study population and cultural context of the sample. For instance, Copp et al. [36] included young adults and the study was conducted in the USA whereas the current study included young and older adults from SSA. One potential justification is that unlike supportive attitudes that leads to tolerance for violence [35], having an attitude that is unsupportive of domestic violence can reduce the tendency for the normalization of violence. Hence, such women would be empowered to exit intimate relationships that exhibit signs of potential violence. Having an unsupportive attitude towards violence has the potential to encourage women to resist the traditional gender roles that promote male dominance and control, which are often precursors to IPV. That is, by standing up against oppressive behaviors, women can create a more egalitarian dynamic, thereby reducing the likelihood of experiencing IPV [35].

### Implications for policy and practice

The findings of this study underscore the importance of women’s empowerment in combatting IPV in SSA. It highlights a need for sub-Saharan African countries to accelerate efforts to improve women’s attitudes to domestic violence, enhance their social independence and decision-making. Practically, this can be achieved through the implementation and strengthening of existing IPV advocacy and economic livelihood initiatives that would guarantee the autonomy/social independence of women in SSA.

### Strengths and limitations

This is arguably the first study in SSA to assess association between SWPER and IPV. Hence, it provides valuable insights into the current body of women’s empowerment and IPV prevention. Also, the study used a large data set that has the statistical power to allow for the extrapolation of the findings to the wider population. Nevertheless, the inherent limitation of the SWPER lies in the point that it is only applicable to partnered/married women. Additionally, the SWPER excludes some important empowerment variables such as women’s ownership status. The self-reported nature of the data also lends its way to the potential recall bias. Furthermore, the DHS adopted a cross-sectional design and this limits the study's ability to draw causal inferences.

## Conclusion

Our study has shown that the three dimensions of SWPER significantly predict IPV among women. It is, therefore, imperative for sub-Saharan African countries to adopt initiatives including IPV advocacy programs and economic livelihood empowerment initiatives to enhance women’s attitudes to domestic violence, and improve their social independence/autonomy, and decision-making capacity. The identification of Sierra Leone, Uganda, Liberia, and Angola as hotspots for IPV calls for targeted interventions in these countries to help eliminate IPV among women. These interventions should focus on empowering women to participate in decision-making processes and changing societal attitudes toward violence.

### Supplementary Information

**Additional file 1: Table S1**. Proportion of intimate partner violence per country.

## Data Availability

Data for this study were sourced from Demographic and Health surveys (DHS) and available here: http://dhsprogram.com/data/available-datasets.cfm.
