# Supplementary material for: Association between the survey-based women’s empowerment index (SWPER) and intimate partner violence in sub-Saharan Africa
Source: Reprod Health. 2024 May 10;21:63. doi: 10.1186/s12978-024-01755-8 (PMC11088024; doi:10.1186/s12978-024-01755-8)
Supplement: Supplementary file 1 — Additional file 1: Table S1. Proportion of intimate partner violence per country. [file 12978_2024_1755_MOESM1_ESM.docx]

**Table S1: Proportion of intimate partner violence per country**

| **Country** | **Physical violence** | **Emotional violence** | **Sexual violence** | **Intimate partner violence** |
| --- | --- | --- | --- | --- |
| 1. Angola | 2414 | 24.57 | 6.64 | 34.54 |
| 1. Benin | 10.90 | 29.23 | 6.06 | 32.35 |
| 1. Burundi | 19.03 | 17.25 | 20.32 | 34.30 |
| 1. Cameroon | 19.23 | 22.55 | 6.60 | 31.43 |
| 1. Ethiopia | 16.79 | 20.05 | 8.54 | 27.17 |
| 1. Gambia | 8.70 | 13.41 | 1.77 | 16.69 |
| 1. Liberia | 35.81 | 36.44 | 7.38 | 47.94 |
| 1. Madagascar | 11.70 | 22.95 | 7.19 | 27.98 |
| 1. Mali | 17.91 | 28.05 | 7.75 | 34.01 |
| 1. Malawi | 15.19 | 22.22 | 15.0 | 32.14 |
| 1. Nigeria | 11.47 | 27.10 | 4.60 | 29.77 |
| 1. Rwanda | 19.95 | 24.20 | 10.34 | 31.00 |
| 1. Sierra Leone | 39.00 | 38.97 | 6.30 | 59.98 |
| 1. Chad | 14.68 | 14.60 | 6.73 | 22.06 |
| 1. Tanzania | 26.57 | 28.04 | 9.63 | 38.02 |
| 1. Uganda | 22.99 | 30.81 | 16.88 | 41.55 |
| 1. South Africa | 8.56 | 10.61 | 3.22 | 14.95 |
| 1. Zambia | 20.66 | 22.18 | 11.09 | 32.61 |
| 1. Zimbabwe | 15.90 | 25.22 | 9.58 | 32.37 |
| **All countries** | **17.96** | **24.31** | **8.84** | **32.04** |
